# Investigation progresses of rare earth complexes as emitters or sensitizers in organic light-emitting diodes

**DOI:** 10.1038/s41377-022-00866-w

**Published:** 2022-06-10

**Authors:** Shuaibing Li, Liang Zhou, Hongjie Zhang

**Affiliations:** 1 State Key Laboratory of Rare Earth Resource Utilization, Changchun Institute of Applied Chemistry, Chinese Academy of Sciences, 130022 Changchun, China; 2 University of Science and Technology of China, 230027 Hefei, China; 3 Department of Chemistry, Tsinghua University, 100084 Beijing, China

**Keywords:** Organic LEDs, Photonic devices

## Abstract

Due to unique photo-physical characteristics, rare earth (RE) complexes play important roles in many fields, for example, telecommunications, life science, and organic light-emitting diodes (OLEDs). Especially, thanks to narrow emission bandwidth and 100% theoretical internal quantum efficiency (IQE), the study of RE complexes in the electroluminescence field has been a hot research topic in recent 30 years. As a leading technology in solid-state light source fields, OLEDs have attracted great interest from academic researchers and commercial endeavors. In the last decades, OLED-based products have trickled into the commercial market and developed quickly into portable display devices. Here, we briefly introduce the luminescent characteristics and electroluminescent (EL) study of RE complexes in material synthesis and device design. Moreover, we emphatically reveal the innovative application of RE complexes as sensitizers in OLEDs. Through experimental validation, the application of RE complexes as sensitizers can realize the complementary advantages of RE complexes and transition metal complexes, leading to significantly improved performances of OLEDs. The application of RE complexes as sensitizers provides a new strategy for designing and developing novel high performances OLEDs.

## Abstract (web summary)

Investigation Progresses of Rare Earth Complexes as Emitters or Sensitizers in Organic Light-Emitting Diodes

## Graphical abstract

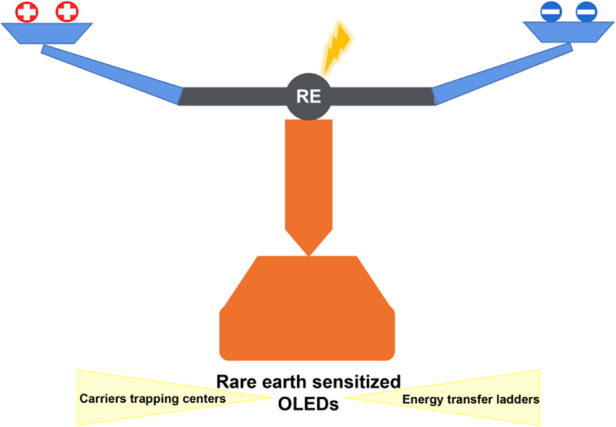

## Introduction

**Fig. 1:**
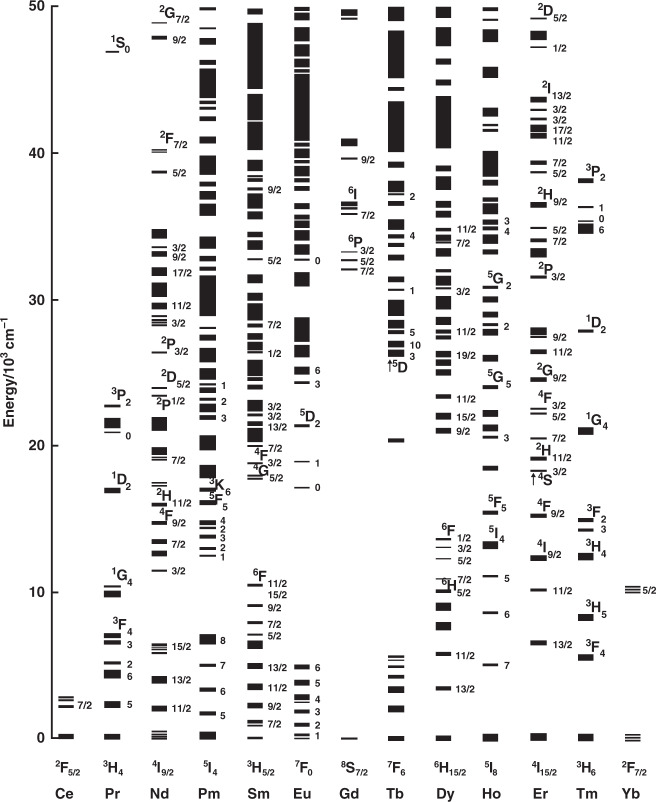
Partial energy levels diagram of RE ions doped in a low-symmetry crystal (LaF3). Reprinted from [3], with the permission of AIP Publishing

Rare earths (REs) are composed of 17 elements in the periodic table. Except for the four elements with atomic number 21, 39, 57, and 71, all other 13 elements have incompletely filled 4 f shells screened by 5s^2^5p^6^ sub-shells^1,2^. As shown in Fig. 1, the unique electronic configure generates rich electronic levels, which endows these 13 RE elements with intricate optical and electromagnetic properties^3^. The energies of these electronic levels are clearly delimited because of the shielding effect of 5s^2^5p^6^ sub-shells, and they are seldom disturbed by external chemical environments. As a corollary, this offers an essential advantage and irreplaceable role for RE complexes in the lighting field. One of the milestones in RE luminescence is the discovery of emissive material Y_2_O_3_:Eu(III), which is the beginning of phosphors used in cathode-ray tubes (CRTs) and fluorescent lamps. Other findings are neodymium Yttrium Aluminium Garnet (YAG) lasers in 1964 and erbium-doped optical fibers in 1987^4,5^. In the mid-seventies, researchers perceived luminescent coordination compounds and proposed Eu(III) and Tb(III) complexes as bioprobes^6^. Furthermore, the design of RE complexes based organic light-emitting diodes (OLEDs) has become another major drive in the expansion of RE coordination compounds^7–9^.

Starting with the demonstration of organic electroluminescent (EL) device by C. W. Tang, OLEDs have been successfully commercialized through three decades of development and honored as the most promising candidate of next-generation display and lighting technology due to high image quality, auto-emitting, flexibility and other merits^10–14^. Actually, the success of OLEDs in the commercial field should profit from the huge boosts of device efficiency and stability. For example, the lifetime of OLEDs at typical brightness levels far surpasses the average lifetime of state-of-the-art mobile phones. Furthermore, the electric-photo conversion efficiency of OLEDs has closed to the theoretical limit by utilizing light extraction technology. Despite enormous progresses of OLEDs technology have been made, there are still inextricable issues that impede the further development of OLEDs^15–17^. One challenge is realizing high efficiency at high brightness level: the difference in carriers’ mobility may reduce carriers recombination probability within emissive layer (EML), thus resulting in low excitons yield. Meanwhile, excess carriers will cause exciton-polar quenching. In addition, due to unbalanced distribution, carriers’ recombination zone is generally very narrow. With increasing current density, exciton density increases rapidly along with severe excitons quenching. In another layout, with the applications of 8 K televisions (7680×4320 dpi) and telemedicine in the future, ultrahigh definition with high color saturation also becomes an urgent requirement in the development of OLEDs.

As we know, RE complexes have line-like emission spectra because of intra-atomic transitions in 4f shell, which establishes outstanding color purity. Simultaneously, the narrow emission spectra are not perturbed by the surrounding environment in view of the shielding effect of external 5s5p electrons. Based on the tremendous transition states of RE ions, emission spectra of RE complexes overlap the scope of ultraviolet (UV), visible light and near infrared (NIR)^18,19^. Moreover, RE complexes can realize 100% internal quantum efficiency (IQE) benefiting from energy transfer from triplet excitons to RE ions^20,21^. According to the aforementioned advantages, RE complexes have been considered as ideal luminescent materials. In recent decades, abundant endeavors have been paid for synthesizing new complex structures, investigating their luminescent mechanisms and expanding their applications^22–24^. For OLEDs, the introduction of RE complexes can improve color saturation and offer theoretically 100% IQE. Although these merits show bright dawn for RE complexes based OLEDs, there are still some issues hindering the practical application of RE complexes in OLEDs^25,26^. One is relatively lower EL efficiency compared with their photoluminescent (PL) efficiency, which is responsible for severe non-radiative relaxation attributed to long exciton lifetime and poor charge transport. Another issue is the poor film-forming property of most RE complexes due to their special molecular structures.

In this review, we firstly introduce the luminescent characteristics of RE complexes. Secondly, the EL mechanisms of RE complexes and their advances in OLEDs are summarized. Then, the emphasis is placed on the discussions of the application of RE complexes as sensitizers in OLEDs, including sensitizing mechanisms and the development of RE complexes sensitized OLEDs. At the end, the prospects and challenges of RE complexes sensitized OLEDs are presented.

## Luminescent characteristics of RE complexes

The luminescent characteristics of RE complexes are associated with the unique electronic structures of RE ions. RE ions have an unfilled 4f shell, where electronic configuration is 4f^n^5s^2^5p^6^ (0 ≤ *n* ≤ 14). In view of the shielding effect of electrons located in 5s and 5p orbitals, the energies of electronic levels vary negligibly with the change of chemical environment, leading to sharp emission spectra (<10 nm) and high color purity^27^. At the same time, about 30,000 spectral lines can be observed in the emission spectra of RE ions benefiting from tremendous transitions, which establish that emissions of RE complexes cover the whole emission range from UV to NIR. Based on the f-f or d-f transitions of electrons in 4f orbitals, RE complexes are separated into two types according to emission wavelength: visible emission (red-emitting Eu(III), orange-emitting Sm(III), yellow-emitting Dy(III), green-emitting Tb(III), blue-emitting Tm(III)) and NIR emission (Nd(III), Er(III) and Yb(III))^28^.

**Fig. 2:**
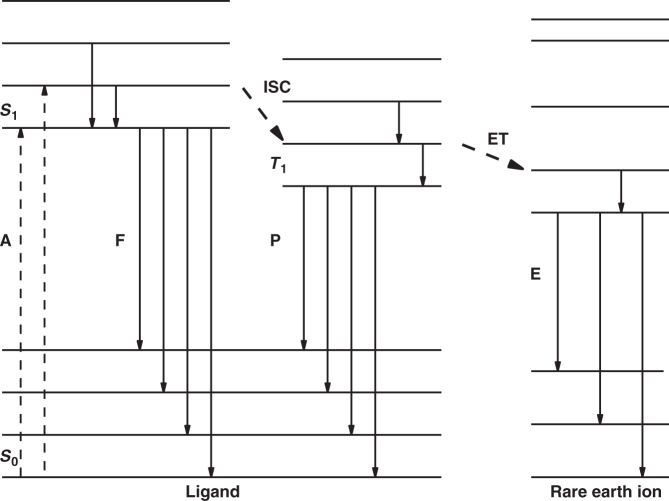
Photo-physical processes in RE complexes. Here A, F, P, and E stand for absorption, fluorescence, phosphorescence and emission, respectively; while ISC and ET are intersystem crossing and energy transfer, respectively

According to the parity selection rule, electric dipole transitions can only occur in energy states with different parity. For f-f transition, the electric dipole transitions of 4f electrons have a relatively low probability due to the same parity of its electronic levels, leading to undesired non-radiative transitions. This seriously prevents the wide application of RE complexes as emitters. As one of landmarks, Weissman and co-workers proposed a strategy of sensitization process, where ligands are introduced to coordinate with RE ions, and the energy of ligands can be transfer to central RE ions^29^. The energy transfer mechanism is described in Fig. 2, which involves three processes: (1) singlet states (S_1_) form on ligands by absorbing excited energy; (2) singlet excitons located on ligands transfer energy to triplet state (T_1_) through intersystem crossing; (3) T_1_ energy excites RE ions via energy transfer and thus generates light through radiative decay. In the process, energy transfer is carried out by the way of resonant coupling between 4f excited states of RE ions and T_1_ of ligands. The corresponding photoluminescent quantum efficiency (PLQE) can be expressed as follows:

1 $${{{\mathrm{\eta }}}}_{{\rm{PL}}} = {{{\mathrm{\eta }}}}_L \ast {{{\mathrm{\eta }}}}_{{\rm{ET}}} \ast {{{\mathrm{\eta }}}}_{{\rm{RD}}}$$

Here, η_PL_ represents PLQE, *η*_L_ is the IQE of ligand, *η*_ET_ is energy transfer efficiency from ligands to RE ions, and *η*_RD_ refers to radiative decay efficiency of RE ions. For specific RE ion, *η*_RD_ is a constant, so the optical properties and energy levels of ligands play crucial roles in the emission process of RE complexes. Moreover, another two energy transfer processes were also reported in some literature^30,31^.

## EL mechanisms and processes of RE complexes

Compared with other display and lighting technologies, the development of OLEDs makes pluralistic display with high quality and low cost possible^32–34^. However, the low efficiency and broad emission spectrum of organic molecules are difficult to satisfy the requirements of up-market displays. This stimulates continuing efforts to develop novel emitter materials. Fortunately, the distinctive luminescent characteristics of RE complexes provide one route to achieve efficient OLEDs with high color purity. RE complexes are well known for their successful optimal applications as up-conversion emitters and fiber amplifiers in optical communications. Since the first report of EL phenomenon from RE complexes, strong interests in the EL study of RE complexes has been ongoing due to rich optical characteristics and high luminescent efficiency^35^. Till the present time, a lot of research activities are focused on investigating RE complexes molecule structures and device performance.

### Basic EL principles of OLEDs

**Fig. 3:**
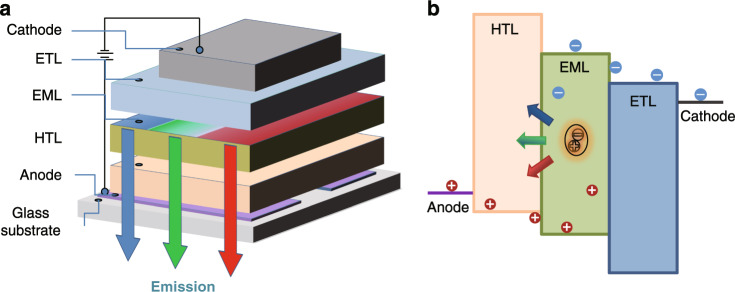
Schematic depictions of OLEDs structure and EL principle. (**a**) Typical OLEDs structure; (**b**) EL principle of OLEDs

As shown in Fig. 3(a), conventional OLEDs are composed of many organic functional layers, which contain carriers transporting layer, EML and other modified layers^36^. These organic functional layers are placed between cathode and anode, where indium-tin-oxide (ITO) is usually utilized as anode material and cathode is composed of metal materials such as Al, Ag, and Mg. Moreover, carrier injection- or block- layers are also employed to regulate the injection and transport of carriers by utilizing small molecules or polymers materials.

The EL principles of OLEDs can be described as follows: under applied electric field, carriers are injected into EML from two electrodes^37,38^. Within EML, holes and electrons recombine and form excitons on emitter molecules, then excitons deactivate with the release of light by radiative transition. The schematic injection, transport and recombination processes are described in Fig. 3b. According to the operation mechanisms, the following EL processes are critical for fabricating high performances OLED:

1. Carriers injection and transport: in OLEDs, due to interface barriers between carrier injection layers and electrodes as well as different organic functional layers, balanced carriers transport is essential for maintaining high carriers utilization probability. Besides, high mobility of carrier transport layers is helpful in lowing drive voltage and enhancing power efficiency (η_p_).
2. Carriers recombination and excitons generation: in guest–host system, carriers hop into EML with the aid of electric field and then trapped by emitter or host molecules along with singlet or triplet excitons formation. Consequently, excitons transition and energy transfer between host and emitter molecules are key attributes. Here, for RE complexes, because of spin-orbital coupling induced by RE ions, all singlet or triplet excitons can realize radiative transitions to obtain 100% theoretical IQE.

### Review on electroluminescence of RE complexes

**Fig. 4:**
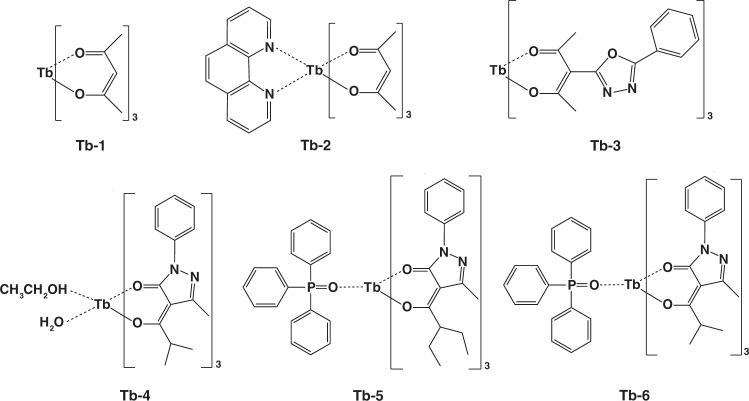
The complexes structures of Tb-1-Tb-6

A starting point for electroluminescence of RE complexes is the demonstration of Tb-1 (Fig. 4) with characteristic green emission by Kido et al. in 1990^35^. They used Tb-1 as both emitter and electron transport material to construct EL devices with the double layers structure of ITO/N,N’-diphenyl- N,N′-(3-methyl phenyl)-1,1′-biphenyl-4,4′-diamine (TPD)/Tb-1/Al. Due to poor carriers transport feature, the maximal brightness of obtained device is only 7 cd m^−2^. Later in 1991, they also reported the electroluminescence features of europium complexes^39^. Even the europium complex based device showed high operation voltage, this work is exciting for developing pure red OLEDs. After these pioneer works, many significant achievements have been realized on synthesizing new RE complexes and optimizing device structure^40–44^. Amongst RE complexes, terbium and europium complexes are the widely concerned materials because of high luminescent efficiency, so this part will discuss the two types of complexes in more detail.

**Fig. 5:**
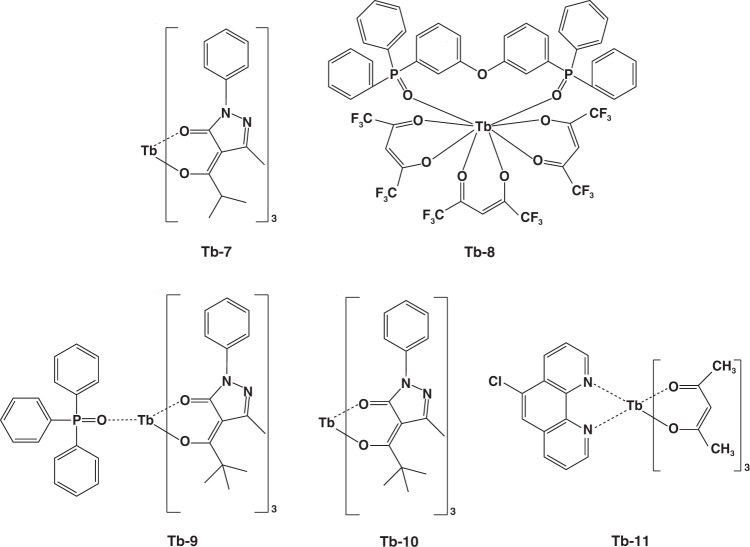
The complex structures of Tb-7-Tb-11

In 1997, Li and co-workers developed two double layers devices by synthesizing Tb-2 (Fig. 4) as emitter, where TPD and poly(9-vinylcarbazole) (PVK) were incorporated to transport hole, respectively^45^. The TPD-based device showed the wide characteristic emission of TPD from 390 to 460 nm, while the PVK-based device exhibited only the emission from Tb^3+^. Benefitting from higher excitons generation probability on emitter molecules, the PVK-based OLEDs obtained maximum brightness of 210 cd m^−2^. In consideration of low carriers transport ability of acetylacetone ligand, Wang et al. designed a terbium(Ш) complex Tb-3 (Fig. 4)^46^. Compared with reference device, the Tb-3 based device obtained higher external quantum efficiency (EQE) of 1.1%, indicating electron-transporting oxadiazole group is helpful in maintaining carriers balance and boosting device performances. In 2003, Xin and co-workers studied the impact of triphenylphosphine oxide (TPPO) ligand on PLQE and carriers mobility of terbium complexes^47,48^. Under the same excited conditions, the relative emission intensity ratio of three complexes (Tb-4, Tb-5, and Tb-6, respectively, Fig. 4) was 1:1.2:2.6, demonstrating TPPO ligand has marked influence on enhancing PLQE. Moreover, Tb-6 exhibited excellent electron transport ability, leading to balanced carriers’ distribution in OLEDs. In view of poor thermal stability of TPPO ligand, Chen and co-workers synthesized 9-(4-tert-butylphenyl)-3,6-bis(diphenylphosphineoxide)-carbazole (DPPOC) ligand^49^. By combining DPPOC together with Tb-7 (Fig. 5), an efficient and stable device was obtained due to the bipolar carriers transport ability and efficient energy transfer of co-doped EML. Based on DPPOC co-doped device structure, a high brightness of 2256 cd m^−2^ with current efficiency (η_c_) of 36.0 cd A^−1^ was achieved. These are the highest values for terbium complex based devices at that time. In 2019, Ilmi et al. reported a novel terbium complex (Tb-8, Fig. 5) by integrating trifluoroacetylacetone (Htfac) and bis(2-(diphenylphosphino)phenyl)ether (DPEPO) as ligands^50^. Based on this complex, the authors prepared and characterized single- and double-EML(s) OLEDs, respectively. Surprisingly, single-EML device based on Tb-8 displayed white emission with a maximum brightness of 1637 cd m^−2^, which is first white OLEDs composed of only tebium(III) complex as emitter. The physicochemical properties of these terbium complexes are depicted in Table 1.

**Table 1:** The physicochemical properties of the representative terbium complexes

Materials	*T*_g_^a^ (°C)	*T*_d_^b^ (°C)	HOMO^c^ (eV)	LUMO^d^ (eV)	PLQY^e^ (%)	*E*_g_^f^ (eV)
Tb-1	140	185	–	–	33	–
Tb-2	–	–	−6.2	−3.3	44	2.9
Tb-3	–	–	–	–	–	–
Tb-4	–	100	−6.4	−3.1	–	3.3
Tb-5	–	300	−6.4	−3.1	–	3.3
Tb-6	–	300	−6.4	−3.1	–	3.3
Tb-7	137	440	−5.4	−1.9	16.7	3.5
Tb-8	176	217	–	–	–	–
Tb-9	–	–	−6.4	−2.6	–	3.9
Tb-10	–	–	–	–	–	–
Tb-11	–	–	−6.3	−3.2	–	3.1

^a^ Glass transition temperature (T_g_)

^b^Decomposiion temperature (*T*_d_)

^c^Highest occupied molecular orbital (HOMO)

^d^Lowest unoccupied molecular orbital (LUMO)

^e^Photoluminescent quantum yield (PLQY)

^f^Energy gap (*E*_g_)

**Fig. 6:**
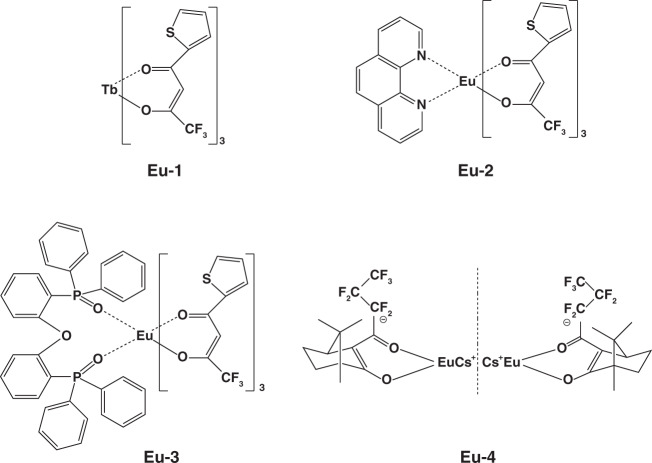
The complexes structures of Eu-1- Eu-4

**Fig. 7:**
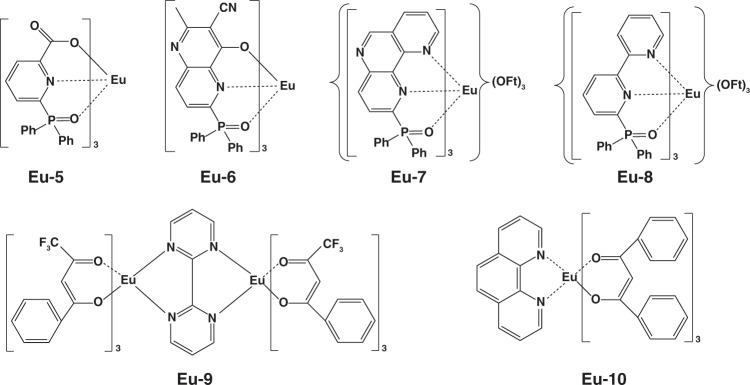
The complexes structures of Eu-5 - Eu-10

Except for terbium complexes, europium(III) complexes were also widely applied in EL devices. In 1991, Kido and co-workers first reported the utilization of europium(III) complex (Eu-1, Fig. 6) as emitting material in OLEDs^39^. However, owing to unsaturated coordination of Eu^3+^, H_2_O molecules in the environment were easily trapped by remaining coordination of Eu-1 molecules, leading to emission quenching of Eu^3+^ and thus poor device performances. Based on Eu-1, Sano and co-workers designed a coordinated europium(III) complex (Eu-2, Fig. 6) by combining the neutral ligand phen^51^. As expected, brightness up to 137 cd m^−2^ was realized by Eu-2 based device. Considering the electron transport characteristic of phen, hole transport type ligand carbazole or diphenylamine was introduced into europium(III) complexes to balance charges’ distribution. Although several examples were reported, the corresponding devices exhibited moderate performances^52,53^. Furthermore, to enhance coordination stability between Eu^3+^ ion and ligands, Xu et al. designed and synthesized Eu-3 (Fig. 6) by utilizing DPEPO as ligand^54^. Compared with TPPO, DPEPO achieved relatively more efficient energy transfer by constructing additional energy transfer route. Finally, a brightness of 632 cd m^−2^ was obtained. In 2015, Zinna et al. constructed a chiral europium complex (Eu-4, Fig. 6) and firstly achieved circularly polarized EL device^41^. Despite this device showed relatively low EQE of 0.0042%, the work proposed a novel strategy for obtaining circularly polarized devices. In 2018, Wei et al. constructed four europium(III) complexes (Eu-5 to Eu-8, Fig. 7) by introducing another diphenylphosphine oxide group (DPPO) into bidentate ligands^55^. In view of stable coordination effect, the complexes exhibited high PLQE of 91% in solid state, which was hopeful for developing highly efficient OLEDs. Ilmi and co-workers reported another new homodinuclear complex (Eu-9, Fig. 7)^56^. Based on this complex, the optimal device displayed a CIE_x,y_ of (0.662, 0.321), approaching the standard red emission (0.670, 0.330). Recently, Utochnikokova and co-workers analyzed the physicochemical properties of specially selected europium complexes containing various ligands and the corresponding OLEDs characteristics, and they discovered that the long lifetime of excited state is an important parameter limiting europium complexes based device performances^57^. The physicochemical properties of these europium complexes are depicted in Table 2.

**Table 2:** The physicochemical properties of the representative europium complexes

Materials	*T*_g_^a^ (°C)	*T*_d_^b^ (°C)	HOMO^c^ (eV)	LUMO^d^ (eV)	PLQY^e^ (%)	*E*_g_^f^ (eV)
Eu-1	180	230	–	–	–	–
Eu-2	245	334	−6.3	−3.3	52	3.0
Eu-3	93	317	–	–	55	–
Eu-4	–	–	–	–	–	–
Eu-5	–	414	–	–	81	–
Eu-6	–	450	–	–	91	–
Eu-7	–	390	–	–	91	–
Eu-8	–	314	–	–	82	–
Eu-9	–	302	–	–	54	–
Eu-10	–	180	−5.9	−2.9	–	–

^a^Glass transition temperature (*T*_g_)

^b^Decomposition temperature (*T*_d_)

^c^Highest occupied molecular orbital (HOMO)

^d^Lowest unoccupied orbital (LUMO)

^e^Photoluminescent quantum yield (PLQY)

^f^Energy gap (*E*_g_)

**Fig. 8:**
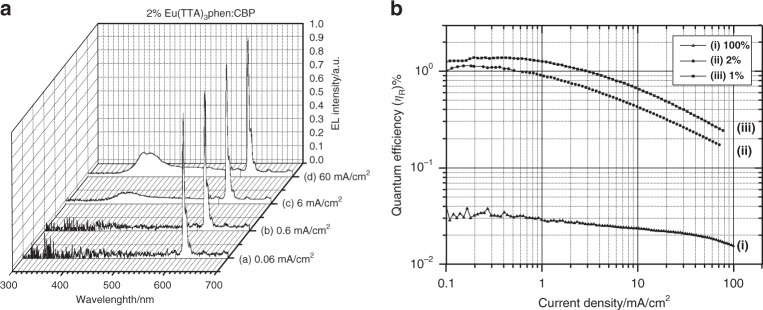
The EL features of Eu-2 based devices. (**a**) EL spectra. (**b**) EQE-current density (EQE*-J*) characteristics. Reprinted from [58], with the permission of AIP Publishing

Besides material synthesis, the design of device structure plays equally key role for developing RE complexes based OLEDs. In 2000, Adachi and co-workers investigated the EL mechanisms of europium complex based multi-layers device composed of ITO/TPD/Eu-2:4,4’-bis(carbazol-9-yl)biphenyl (CBP)/2,9-dimethyl-4,7-diphenyl-1,10-phenanthroline (BCP)/Tris(8-hydroxy-quinolinato)aluminium (Alq_3_)/Mg:Ag (Device-1, Table 3)^58^. In the PL spectrum of EML, CBP emission demonstrated no energy transfer appeared between CBP and Eu-2 molecules. Nevertheless, as shown in Fig. 8a, the device showed pure Eu-2 emission at low current density, which implied that direct carriers trapping on Eu-2 molecules was the dominant EL mechanism. Before long, Capecchi et al. systematically discussed the effect of device construction on EL performances by utilizing Tb-9 (Fig. 5) as emitter^40^. For single-layer OLEDs consisting of ITO/Tb-9/Mg:Ag, EL efficiencies were positively correlated with EML thickness. Then, double layers device was fabricated by employing TPD and 3-(4-biphenylyl)-4-phenyl-5-(4-tert-butylphenyl)-1,2,4-triazole (TAZ) or Alq_3_ as hole- and electron-transport materials, respectively. Nevertheless, this double layers device exhibited lower efficiency because of unbalanced carriers transport. To improve carriers balance, they further designed four layers device (Device-2, Table 3). Due to decreased hole injection barrier, this device achieved the maximum brightness of 2000 cd m^−2^ and η_c_ of 14 cd A^−1^, which were once the best performances for RE complex-based OLEDs. Then, Thorne and co-workers investigated energy transfer mechanisms of Tb-10 (Fig. 5) with a excited state lifetime of 160 μs^59^. They discovered that high PLQE still could be retained at high excitation energy. So, they believed that the decisive factor of the roll-off in efficiency for Tb-10-based OLEDs was not triplet-triplet annihilation (TTA) but triplet-polar quenching (TPQ). Our group investigated the dominant EL mechanisms based on Eu-2 doped CBP system in 2007^60^. Through analyzing the evolution of carriers’ distribution within EML, we discovered that only electrons’ trapping exists in Eu-2 doped CBP system, thus leading to unbalanced carriers’ distribution on Eu-2 molecules. With the increase of voltage, the change of carriers’ distribution influences strongly EL processes. As a result, dominant EL mechanism transforms substantially from carriers’ trapping into energy transfer. In the next year, we further investigated the effects of carriers’ distribution within EML on device efficiency^42^. For Eu-2 doped CBP system, EL efficiency monotonously increases with the increase of holes injection. This phenomenon is attributed to holes accumulation within EML, leading to improved carriers balance on Eu-2 molecules. To further improve carriers balance on Eu-2 molecules, we gently decreased the injection of electrons into EML by changing the thickness of cathode. As a result, with the decrease of electrons injection, EL efficiency subsequently increased and then decreased rapidly. Therefore, we concluded that carriers balance on emitter molecules was the critical condition to achieve high EL efficiency. In 2016, Yu et al. utilized carriers confinement method to improve carriers balance^61^. Firstly, carriers transport materials with large energy gap were chosen to restrain triplet excitons diffusion. Then, they employed a double-EML structure to confine carriers’ recombination zone within EMLs. Lastly, P-doped holes transport layer was employed to enhance holes injection. Finally, they fabricated EL device with the structure of ITO/MoO_3_/4,4′,4prime″-tri(9-carbazoyl)triphenylamine (TcTa)/Tb-7 (Fig. 5):di(9H-carbazol-9-yl)(phenyl)phosphine oxide (DCPPO)/Tb-7:DPPOC/tris-(3-(3-pyridyl)mesity)borane (3TPYMB)/LiF/Al (Device-3, Table 3), and the device achieved the maximum η_c_ of 57 cd A^−1^, and EQE of 14.8%. These performances were amongst the best-reported values of terbium complex-based devices. In 2017, Zhao et al. constructed a device composed of ITO/MoO_3_/1,3-bis(9-carbazoly)benzene (mCP)/mCP:(1,3,5-triazine-2,4,6-triyl)tris(benzene-1,3-diyl)tris(dipenylphosphine oxide) (POT2T):Eu-10 (Fig. 7)/POT2T/4,7-diphenyl-1,10-phenanthroline (Bphen)/LiF/Al (Device-4, Table 3), which displayed low turn-on voltage of 2.5 V^62^. In 2019, Girotto and co-workers reported co-host structure composed of TcTa and 2,2’-(1,3-phenylene)bis[5-(4-tert-butylphenyl)-1,3,4-oxadiazolespirobifluorene (OXD-7), and fabricated a series of solution-processed OLEDs^63^. Due to relatively balanced carriers’ distribution, the turn-on voltage of the optimal device (Device-5, Table 3) is reduced to 2.3 V, and the brightness is up to 6365 cd m^−2^. Especially, negligible efficiency roll-off was also achieved.

**Table 3:** The EL performances of the representative RE complexes based devices

Device	EML	*V*_turn-on_ (V)	*B*^a^ (cd m^−2^)	η_c_^b^ (cd A^−1^)	η_p_^c^ (lm W^−1^)	EQE^d^ (%)	Refs.
Device-1	Eu-2:CBP	–	>500	–	–	1.4	58
Device-2	Tb-9	10	>2000	2.63	14	–	40
Device-3	Tb-7:DCPPO/Tb-7:DPPOC	3.4	2784	52.00	57.00	14.8	61
Device-4	Eu-10:mCP:POT2T	2.5	330	4.94	5.51	3.11	62
Device-5	Tb-C:TcTa:OXD-7	2.3	6365	17.16	–	–	6
Device-6	Ce-1:CBP	–	13 μW cm^−2^	–	–	–	64
Device-7	Nd-1:TcTa:SPPO13	–	–	–	–	0.034	65
Device-8	Yb-1:Mcp	–	390 μW cm^−2^	–	–	0.17	66
Device-9	Yb-2	–	–	–	–	0.21	67

^a^Maximum brightness (B)

^b^Maximum current efficiency (η_c_)

^c^Maximum power efficiency (η_p_)

^d^Maximum external quantum efficiency (EQE)

**Fig. 9:**
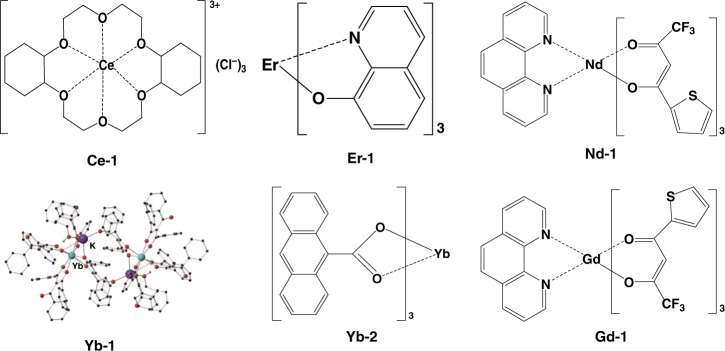
The complex structures of Ce-1, Er-1, Nd-1, Yb-1, Yb-2 and Gd-1

Besides terbium and europium complexes, some OLEDs containing other RE complexes were also reported^64–66^. Cerium ion is easily quenched by regular ligands and surrounding molecules, so it is a burdensome task to synthesis high PLQE cerium complexes, leading to few reports on cerium complexes. Yu and co-workers investigated the PL and EL properties of Ce-1 (Fig. 9), and fabricated Ce-1 based OLEDs (Device-6, Table 3)^67^. They found the device displayed mainly CBP emission when Ce-1 concentration was less than 3 wt%; with increasing Ce-1 concentration, the device did not work because of severe excitons quenching. Some RE complexes with NIR emission, such as neodymium complexes, erbium complexes and ytterbium complexes, were also investigated^68–70^. In 2019, Shahalizad et al. fabricated Nd-1 (Fig. 9) based OLEDs by utilizing solution processing method^71^. Co-host EML structure was utilized in designed devices. The optimal device composed of ITO/PEDOT:PSS/TcTa:2,7-bis(diphenylphosphoryl)-9,9’-spirobifluorene (SPPO13):Nd-1/2,2′,2″-(1,3,5-benzinetriyl)-tris(1-phenyl-1-H-benzimidazole) (TPBi)/LiF/Al (Device-7, Table 3) achieved the maximum EQE of 0.034%, which is as high as that of evaporation processing NIR OLEDs, and it is one of the best solution processing NIR OLEDs. In 2018, Nagata et al. utilized Er-1 (Fig. 9) as NIR phosphor to produce triplet excitons via singlet fission^72^. According to their report, there was not marked overlap between the absorption spectrum of Er-1 and the emission spectrum of 5,6,11,12-tetraphenylnaphthacene (rubrene). So, Fӧreter energy transfer between Er-1 and rubrene molecules was inefficient. Triplet energy induced through singlet fission could be finally transferred to Er^3+^ owing to relatively lower triplet energy of rubrene. Therefore, the overall excitons production efficiency reached 100.8%, which breaks the theoretical limit of 100%. Due to the unique emission at 980 nm and monochromaticity, ytterbium complexes are regarded as the most excellent NIR emitting RE complexes, and the reports of ytterbium complexes increase gradually in recent years. In 2014, Reid and co-workers designed a new ytterbium complex (Yb-1, Fig. 9) by utilizing β-diketone LH as ligand^73^. In solid state, Yb-1 had the excited lifetime of 42 μs and realized the PLQE of 3.9%. The corresponding device (Device-8, Table 3) displayed Yb^3+^ characteristic emission with the maximum EQE of 0.17%. Utochnikova et al. reported another ytterbium complex (Yb-2, Fig. 9) with PLQE of 1.5%, and the corresponding non-doped devices (Device-9, Table 3) realized the maximum EQE of 0.21%^74^. The physicochemical properties of these representative RE complexes are shown in Table 4. Besides the utilization in EL devices, some ytterbium complexes have been widely used in NIR imaging field. Among them, ytterbium complexes with fluorinated porphyrins and porpholactones ligands exhibited extraordinary quantum yields and stability, and several meaningful works on the application of ytterbium complexes as NIR probes have been reported by Zhang et al.^75–77^.

**Table 4:** The physicochemical properties of the representative other RE complexes

Materials	*T*_g_^a^ (°C)	*T*_d_^b^ (°C)	HOMO^c^ (eV)	LUMO^d^ (eV)	PLQY^e^ (%)	*E*_g_^f^ (eV)
Ce-1	3.4	310	–	–	55	–
Er-1	–	–	−5.3	−2.2	5	3.1
Nd-1	–	–	−5.4	−2.5	12	2.9
Yb-1	–	300	–	–	3.9	–
Yb-2	–	350	−4.3	−2.5	1.5	2.8
Gd-1	–	–	−6.3	−3.3	–	3.0

^a^Glass transition temperature (T_g_)

^b^Decomposition temperature (T_d_)

^c^Highest occupied molecular orbital (HOMO)

^d^Lowest unoccupied molecular orbital (LUMO)

^e^Photoluminescent quantum yield (PLQY)

^f^Energy gap (*E*_g_)

## Application of RE complexes in OLEDs as sensitizers

As mentioned above, significant efforts have been paid on the development of RE complexes based OLEDs. So far, the highest PLQE of RE complexes has reached 100%, while the highest EQE of RE complexes based OLEDs is only 15%^61^. This arises from serious excitons quenching effect. Because the f-f transitions are partially forbidden, excitons lifetimes of RE complexes (from μs to ms range) are usually much longer than those of transition metal complexes or thermally activated delayed fluorescence molecules (in μs range), which speeds up the efficiency roll-off of OLEDs. Additionally, another impediment is the poor carriers transport properties of most RE complexes caused from molecule configurations. Consequently, the development of RE complexes as emitters in OLEDs still remains challenge.

Among various approaches of improving OLEDs performances, we have proposed a strategy of using RE complexes as sensitizers in OLEDs and have successfully constructed a series of highly efficient RE complexes sensitized OLEDs. Considering wide energy gaps, RE complexes molecules form deep carriers trapping centers and retard carriers’ migration. In other words, RE complexes possess the ability of regulating carriers transport and maintaining carriers balance on emitter molecules. Moreover, through rationally choosing RE complexes with well-matched energy levels, RE complexes molecules can also play another role of energy transfer ladders, thus leading to high excitons utilization efficiency. Consequently, the strategy of using RE complexes as sensitizers in OLEDs smartly turns the shortcomings (wide energy gap and long excited lifetime) of RE complexes into their key advantages. The specific sensitizing mechanisms and current investigations of RE complexes sensitized OLEDs will be presented as follows.

### Sensitizing mechanism

Compared with fluorescent OLEDs, one shortcoming of phosphorescent OLEDs is their severe efficiency roll-off phenomenon owing to slow triplet excitons decay of phosphorescent materials^78–81^. According to previous investigations, TTA and TPQ are concluded as the essential reasons contributed to efficiency roll-off^82,83^. To deeply analyze excitons quenching mechanisms, triplet excitons density and excitons quenching pathways were described as follows^15,84^:

2 $$\frac{{{\rm{d}}n_T(t)}}{{{\rm{d}}t}} = \frac{{n_T(t)}}{{\tau _T}} - \frac{1}{2}k_{TT}n_T(t)^2$$

3 $$T_1 + T_1 \to X\left\{ {\begin{array}{*{20}{c}} { \to S_n + S_0 \to S_1 + S_0} \\ { \to T_n + S_0 \to T_1 + S_0} \end{array}} \right.$$

Here, *τ*_*T*_ and *k*_*TT*_ are triplet exciton lifetime and the rate of TTA, respectively, *T*_*n*_, *T*_1_, and X represent high-lying triplet state, lowest triplet state and intermediate state, respectively, and *S*_*n*_, *S*_1_, and *S*_0_ are high-lying single state, lowest single state and ground state, respectively. In Eq. (2), the last term presents biexcitonic annihilation, which demonstrates that TTA rate increases non-linearly with increasing current density. During TTA process, all triplet excitons will go through the above two pathways (Eq. (3)), and then be transferred back to S_1_, T_1_ or S_0_. Eventually, part triplet excitons will be wasted, resulting in low excitons utilization efficiency. For OLEDs, another problem is the unbalanced carriers’ distribution within EML(s), which leads to undesirable carriers’ accumulation near interface and thus narrow recombination zone. In addition, within guest-host system, emitter molecules usually act as carrier trapping sites, and excess carriers will be trapped by emitter molecules because of different carriers’ mobility. With increasing applied electric field, excess carriers within EML lead also to significant TPQ. Moreover, excess carriers can cause increasing Joule heat within OLEDs thus accelerating material aging, which is seriously harmful for improving the stability of devices. Consequently, balanced carriers’ distribution and broadening recombination zone are essential for solving efficiency roll-off and stability problems of OLEDs.

A key for designing efficient OLEDs is to understand carriers transport characteristics. According to our investigations, carriers’ trapping on emitter molecules followed by direct excitons formation is one of the dominant EL mechanisms of OLEDs^85,86^. Generally, EML is composed of host and emitter materials, where host material is preferentially electron or hole transport. Under electric field, carriers are injected into EML via carrier transport layers, and part carriers are trapped by emitter molecules along with excitons formation. Due to single carrier transport characteristic of most host materials, holes or electrons will become the multiple carriers within recombination zone, resulting in unbalanced carriers’ distribution. Moreover, redundant carriers will exacerbate excitons quenching process by the way of TPQ. To balance carriers’ distribution within EML, we have recently put forward the innovative application of RE complexes as sensitizers. Through utilizing ternary trace doping technology, RE complexes were introduced into EML to construct RE complexes sensitized OLEDs. In RE complexes sensitized OLEDs, RE complexes molecules store superfluous carriers, and then the trapped carriers can be continuously released and transferred into emitter molecules through intermolecular hopping with increasing applied electric field. In this extent, excitons quenching probability caused by excessive carriers will be decreased, while local carriers balance on emitter molecules can be realized. It is particularly worth mentioning that trapping effect of RE complexes has no negative influence on η_p_ owing to the trace doping of RE complexes. Additionally, RE complexes molecules with appropriate triplet energy play also the role of energy transfer ladders and thus accelerate energy transfer towards emitter molecules, resulting in faster excitons radiative decay and mitigated excitons quenching. Consequently, efficiency roll-off problem is relieved to some extent in RE complexes sensitized OLEDs, and excellent EL performances were realized.

### RE complexes sensitized OLEDs

**Fig. 10:**
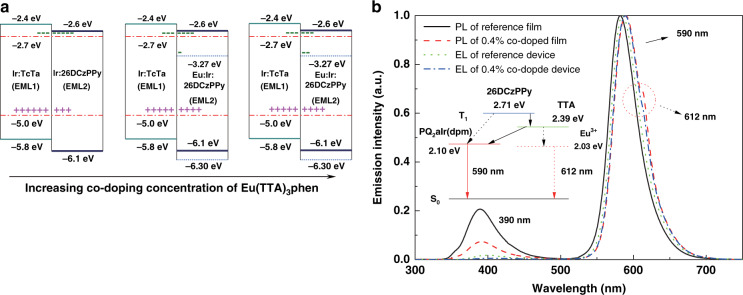
**Carriers**’ **distribution characteristics in Eu-2 sensitized devices and the corresponding PL and EL spectra.** **a** Carriers’ distribution within the EMLs of sensitized devices at different co-doped concentrations. **b** PL spectra of reference and 0.4 wt% co-doped films and EL spectra of the corresponding devices. Insert: Schematic representation of energy transfer processes within the co-doped EML. Reprinted with permission from [87]. Copyright {2015} American Chemical Society

In 2015, RE complexes sensitized OLEDs were firstly reported by Zhou et al.^87^. This pioneer work employed the wide band gap Eu-2 as sensitizer to control electrons accumulation on Irdium(III)bis(2-phenylquinoly-N,C2’)-dipivaloylmethane (PQ_2_Ir(dpm)) molecules. The designed device has the structure of ITO/ Di-[4-(N,N-di-p-tolyl-amino)-phenyl]cyclohexane (TAPC)/PQ_2_Ir(dpm):TcTa/Eu-2:PQ_2_Ir(dpm):2,6-bis(3-(9H-carbazol-9-yl)phenyl)pyridine (26DCzPPy)/1,3,5-tri(m-pyrid-3-yl-phenyl)benzene (TmPyPB)/LiF/Al (Device-10, Table 5). In these devices, the stepwise HOMO and LUMO levels alignments facilitate carriers’ injection and transport. As expected, sensitized devices displayed relatively higher performances and better color purity. As described in Fig. 10, in reference devices, electrons were the multiple carriers within EML2 because of the excellent electron transport ability of 26DCzPPy, which caused carriers’ balance on PQ_2_Ir(dpm) molecules to grow worse. When trace Eu-2 molecules were co-doped into EML2, some electrons were preferentially trapped by Eu-2 molecules because of lower LUMO level, thus restraining electrons accumulation on PQ_2_Ir(dpm) molecules. Therefore, the improvement of carriers’ balance on PQ_2_Ir(dpm) molecules was responsible for boosted EL performances of sensitized devices. In addition, with decreasing electron transport, recombination zone was shifted to cathode and broadened. Therefore, decreased excitons density within EML was helpful in mitigating excitons quenching and delaying efficiency roll-off. Furthermore, though analysis of photo-physical characteristics, Eu-2 molecules played also the role of energy transfer ladders between 26DCzPPy and PQ_2_Ir(dpm) molecules due to well-matched triplet energy. Consequently, the facilitated energy transfer further enhanced the utilization efficiency of excitons energy, resulting in elevated EL efficiency and improved color purity of these sensitized devices. This work presents a novel strategy to broaden the application scope of RE complexes in OLEDs.

**Table 5:** The key properties of some representative RE complexes sensitized devices

Device	Sensitizer/emitter	*V*_turn-on_ (V)	*B*^a^ (cd m^−2^)	η_c_^b^(EQE)^c^ (cd A^−1^)	η_p_^d^ (lm W^−1^)	η_c_^e^(EQE^f^) (cd A^−1^)	Refs.
RD^g^-10	/PQ_2_Ir(dpm)	3.3	73993	43.34(15.4%)	36.65	40.44(14.4%)	92
Device-10	Eu-2/PQ_2_Ir(dpm)	3.0	100870	58.08(21.0%)	60.79	51.94(18.5%)	87
RD-11/12	/PQ_2_Ir(dpm)	3.1	113003	51.26(19.5%)	51.40	46.43(17.7%)	93
Device-11	Tb-11/PQ_2_Ir(dpm)	2.8	145071	64.87(24.7%)	69.11	59.70(22.7%)	88
Device-12	Gd-1/PQ_2_Ir(dpm)	2.8	130774	55.89(21.3%)	51.45	54.18(20.6%)	88
RD-13	/Ir(tfmppy)_2_(tpip)	2.8	55829	92.50(22.3%)	87.12	89.57(21.6%)	94
Device-13	Tb-11/Ir(tfmppy)_2_(tpip)	2.9	89266	114.23(27.6%)	101.92	113.71 (27.5%)	89
RD-14	/Ir1	3.3	162879	82.68(27.0%)	71.00	75.98(24.8%)	95
Device-14	Tb-11/Ir1	3.2	183729	89.42(27.9%)	87.74	74.23(23.2%)	90

^a^Maximum brightness (*B*)

^b^Maximum current efficiency (η_c_)

^c^Maximum external quantum efficiency (EQE)

^d^Maximum power efficiency (η_p_)

^e^Current efficiency (ηc) at the brightness of 1000 cd m^−2^

^f^External quantum efficiency (EQE) at the brightness of 1000 cd m^−2^

^g^Eeference device (RD)

**Fig. 11:**
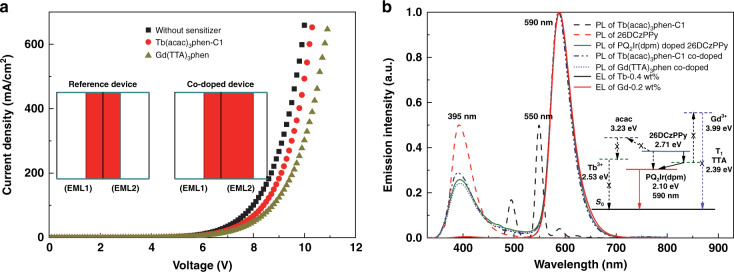
**The EL profiles of PQ**_**2**_**Ir(dpm) based devices.** **a** Current density–Voltage (*J–V*) characteristics of the electron-only devices. Inset: Comparison diagram of the relative recombination zones. **b** Normalized PL spectra of Tb-11 film, 26DCzPPy film, 4 wt% PQ_2_Ir(dpm) doped 26DCzPPy film, 0.4 wt% Tb-11 co-doped 26DCzPPy film, as well as 0.2 wt% Gd-1 co-doped 26DCzPPy film. Insert: Schematic representation of energy transfer processes. Reprinted from [88], with the permission of Royal Society of Chemistry

**Fig. 12:**
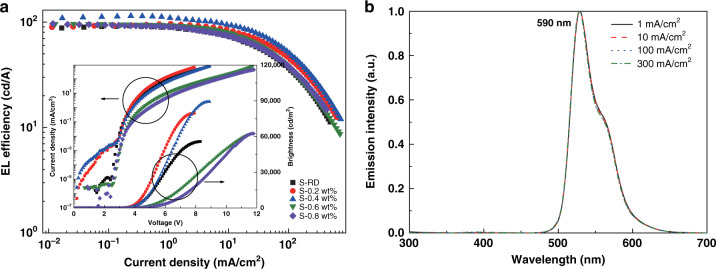
The EL characteristics of Ir(tfmppy)_2_(tpip based devices at different co-doped concentrations. **a** Efficiency–current density (*η–J*) characteristics. Insert: current density–brightness–voltage (*J–B–V*) characteristics. **b** Normalized EL spectra. Reprinted from [89], with permission from Elsevier

**Fig. 13:**
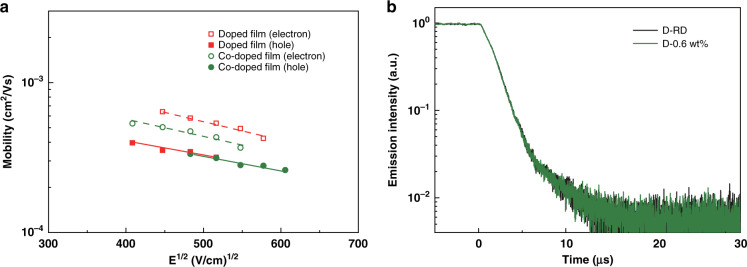
**The carriers transport characteristics and EL decay profiles of Ir1 based devices.** **a** Carriers mobilities of doped films and Tb-11 co-doped films. **b** Transient EL decay profiles of reference device and sensitized device. Reprinted from [90], with the permission of Royal Society of Chemistry

In 2017, Cui et al. utilized terbium complexes (Tb-11, Fig. 5) and gadolinium complexes (Gd-1, Fig. 9) as sensitizers and constructed red-emitting OLEDs based on iridium complex^88^. Even if current density rose to 300 mA cm^−2^, sensitizer emission was still invisible for the sensitized devices, which verified the efficacy of the two RE complexes as sensitizers. Through contrastive analysis, as shown in Fig. 11, Tb-11 exhibited a relatively stronger ability in improving carriers balance, while Gd-1 produced relatively greater effect on energy transfer toward emitter molecules. Meanwhile, more balanced carriers’ distribution on emitter molecules was realized in Tb-11 co-doped system along with higher carriers’ recombination efficiency and excitons radiative probability. For Gd-1 co-doped system, benefiting from appropriate triplet energies of 26DCzPPy (2.71 eV), ligand TTA (2.39 eV) and PQ_2_Ir(dpm) (2.10 eV), Gd-1 molecules functioned as energy transfer ladders and facilitated energy transfer toward PQ_2_Ir(dpm) molecules. Consequently, compared with reference devices, Gd-1 sensitized devices realized higher efficiencies and brightness. The key properties of two optimal sensitized devices (Device-11 and Device-12, respectively,) are listed in Table 5. To estimate the feasibility of sensitizing system, the application of Tb-11 co-doped system was expanded to green-emitting devices based on well-known green-emitting material bis(4-trifluoromethylphenylpyridine)(tetraphenylimido-diphosphinate)iridium (III) (Ir(tfmppy)_2_(tpip))^89^. Similarly, no Tb-11 emission appeared in EL spectra of these sensitized devices, and 26DCzPPy emission was also suppressed, which is due to decreased carriers recombination probability on 26DCzPPy molecules as well as accelerated energy transfer to Ir(tfmppy)_2_(tpip) molecules. Nevertheless, strong 26DCzPPy emission appeared in PL spectra of both unsensitized film and Tb-11 sensitized film, demonstrating electrons trapping on Tb-11 molecules was dominantly responsible for enhanced EL performances. Finally, as described in Fig. 12, the optimal sensitized device (Device-13, Table 5) obtained the maximum EQE up to 27.6%. In 2019, encouraged by these results, the authors deeply investigated the sensitizing mechanisms of green-emitting OLEDs containing iridium(III) bis(2’,6’-bis(trifluoromethyl)-2,3’-bipyridine) tetraphenylimido-diphosphinate (Ir1)^90^. It was noted that double-EMLs devices showed unmarked improvement in EL performances compared with those of single-EML devices, which demonstrated recombination zone locates mainly within electron dominant EML. Considering narrow recombination zone in electron dominant EML, more complicated interactions between carriers and excitons could be anticipated, so the exploration of carriers’ distribution within EML is more necessary. Through the verification of time-of-flight (TOF) technology shown in Fig. 13, when Tb-11 was co-doped into EML, electron mobility decreased markedly, indicating excess electrons were trapped by Tb-11 molecules owing to its lower LUMO level. Furthermore, the deficient holes-trapping capacity of Tb-11 guaranteed that excitons formation mainly took place on Ir1 molecules. Simultaneously, in double-EMLs structures, Tb-11 co-doped device exhibited similar excitons decay lifetime with that of reference device, demonstrating the co-doping of Tb-11 molecules has no marked influence on energy transfer process. That is to say, the regulation of carriers balance is the major contributor of boosted device performances. As anticipated, based on precise carriers’ regulation, the co-doped device exhibited excellent EQE up to 27.9% (Device-14).

## Conclusions and prospects

Through the review, we have briefly introduced the EL investigations of RE complexes and reviewed the development of RE complexes as emitters and sensitizers in OLEDs. Generally, the wide energy gaps and long excited lifetimes of RE complexes caused poor carriers transport and serious excitons quenching during EL processes, which have become insurmountable obstacles for the development of RE complexes as emitters in OLEDs. Nevertheless, wide energy gap endows RE complexes with carriers trapping function, proceeding to regulate carriers balance within EML. Furthermore, the long excited lifetimes of RE complexes are helpful in facilitating energy transfer toward emitter molecules, leading to enhanced energy utilization efficiency. More specifically, in RE complexes sensitized OLEDs, RE complexes play a crucial role in regulating carriers balance and accelerating excitons transformation, which greatly improved EL efficiency and operation stability of OLEDs. These results signify the great potential of RE complexes as sensitizers in fabricating high performances OLEDs.

Although the sensitizing strategy has made great breakthrough in constructing high performance OLEDs, there are still some challenges. So far, the reported suitable RE complexes as sensitizers are mostly europium(III), terbium(III) and gadolinium(III) complexes, so current bottleneck lies in the design and optimization of new molecule structures with multi-functionalization. Moreover, RE complexes usually display poor long-term stability and film-forming properties. Therefore, how to maintain device stability in this situation is also an important issue. As displayed above, although some profound viewpoints have been discussed, better understanding the detailed sensitizing processes is still needed. Furthermore, in terms of device preparation technology, the development of more precise control technology in device preparation processes will greatly improve the reliability and repeatability of OLEDs. We will face these challenges and believe that RE complexes have a broad application in OLEDs.
